# Comparative genomics of a novel Erwinia species associated with the Highland midge (Culicoides impunctatus)

**DOI:** 10.1099/mgen.0.001242

**Published:** 2024-04-17

**Authors:** Jack Pilgrim

**Affiliations:** 1 Institute of Infection, Veterinary and Ecological Sciences, Faculty of Health and Life Sciences, University of Liverpool, Liverpool, UK

**Keywords:** comparative genomics, *Culicoides*, *Erwinia*, insect, symbiosis

## Abstract

*Erwinia* (Enterobacterales: Erwiniaceae) are a group of cosmopolitan bacteria best known as the causative agents of various plant diseases. However, other species in this genus have been found to play important roles as insect endosymbionts supplementing the diet of their hosts. Here, I describe *Candidatus* Erwinia impunctatus (Erwimp) associated with the Highland midge *Culicoides impunctatus* (Diptera: Ceratopogonidae), an abundant biting pest in the Scottish Highlands. The genome of this new *Erwinia* species was assembled using hybrid long and short read techniques, and a comparative analysis was undertaken with other members of the genus to understand its potential ecological niche and impact. Genome composition analysis revealed that Erwimp is similar to other endophytic and ectophytic species in the genus and is unlikely to be restricted to its insect host. Evidence for an additional plant host includes the presence of a carotenoid synthesis operon implicated as a virulence factor in plant-associated members in the sister genus *Pantoea*. Unique features of Erwimp include several copies of intimin-like proteins which, along with signs of genome pseudogenization and a loss of certain metabolic pathways, suggests an element of host restriction seen elsewhere in the genus. Furthermore, a screening of individuals over two field seasons revealed the absence of the bacteria in *Culicoides impunctatus* during the second year indicating this microbe-insect interaction is likely to be transient. These data suggest that *Culicoides impunctatus* may have an important role to play beyond a biting nuisance, as an insect vector transmitting Erwimp alongside any conferred impacts to surrounding biota.

**Impact Statement**

*Erwinia* bacteria are known to cause plant diseases but they can also form diverse relationships with insects. In these interactions, insects may serve as vectors facilitating bacterial transmission, or engage in endosymbiosis, where the bacteria provide them with B vitamins or facilitate nitrogen recycling. This work describes the discovery of *Candidatus* Erwinia impunctatus (Erwimp) associated with the Highland biting midge *Culicoides impunctatus*. Through comparative genomics and field screening of populations across two field seasons, my findings suggest that Erwimp is unlikely to be an endosymbiont of *Culicoides impunctatus*. Instead, it aligns more closely with the majority of other culturable genus members, wherein insects serve as transient hosts. Due to *Culicoides*’ roles in the transmission of veterinary arboviruses and as biting pests, their phytophagous behaviour and potential to transmit plant bacteria is often disregarded. Subsequently, these findings reveal that *Culicoides impunctatus*, traditionally perceived primarily as a pest, may assume multifaceted roles including mediating microbial interactions that could have broader implications for ecosystem health and function.

## Data Summary

Bioinformatic code used for Erwimp genome assembly and downstream comparative analysis doi: 10.6084/m9.figshare.25470235. All raw nanopore reads and *Erwinia* binned DNBseq reads used for assembly and genome polishing are archived in the European Nucleotide Archive (ENA) under ERR11548505, ERR11576402 as part of BioProject PRJEB62526. NCBI accessions for Erwimp are available under OZ024666 (chromosome) and OZ024667 (plasmid). AntiSMASH results, virulence factor and plant gene matrices can be found at https://doi.org/10.6084/m9.figshare.25470091 and https://doi.org/10.6084/m9.figshare.24200700.v1.

## Introduction

Animals and plants establish interactions with bacteria ranging from pathogenic (antagonistic), through to commensal (neutral) and mutualistic (beneficial). Within these categories, insect-microbe interactions are of particular interest due to insect roles as crop pests, insect vectors and pollinators. Certain bacteria are able to persist within cells and specialized tissues of insects (endosymbiosis) which has led to several instances where the fitness of the insect is intrinsically linked with that of the bacteria [1]. Such phenomena have led to a range of phenotypes such as host reproductive manipulation [2], defence against natural enemies [3,4] and diet supplementation [5,6]. In other cases, bacteria have passing interactions with insects, either employing them as vectors on the insects' mouthparts [7] and cuticle surface [8] or by carrying them as gut bacteria [9] where they can be passed on to plants and animals.

An exemplar group of bacteria which embodies this range of interactions with invertebrates are *Erwinia* spp. (Enterobacterales: Erwiniaceae). Endosymbiotic examples include *Erwinia haradaeae* which has evolved as an obligate primary (essential) endosymbiont supplementing *Cinara* aphids with essential B-vitamins and has a reduced genome (~1.1 Mbp) with a relative enrichment of house-keeping genes and those genes relevant for nutritional symbiosis [10]. On the other hand, *Erwinia dacicola* present in the olive fly *Bactrocera oleae* has a more complex lifecycle as an endosymbiont which transitions between an intracellular stage in larval midguts to an extracellular phase in the gut lumen of adults [11] where it is thought to supplement its host with nitrogen through a urease operon [12]. Despite these specialised insect roles, a majority of *Erwinia* are phytopathogens which are in part horizontally transmitted by insects feeding and resting on a primary plant host [13,14]. The most well-studied plant pathogens include *Erwinia amylovora* and *Erwinia pyrifoliae* which can cause devastating disease outbreaks in members of the Rosaceae family, however, other species (e.g. *Erwinia tasmaniensis*) are considered epiphytic non-pathogens [15]. More rarely, *Erwinia* have been isolated from fungi [16] and humans [17,18] where they infrequently cause disease in the latter.

In the absence of ecological and biochemical characterisation of newly identified bacteria, comparative genomics can be used to narrow down ecological niches as well as suggest the nature of interactions with a host. In this study, I use comparisons of existing *Erwinia* genomes encompassing insect endosymbionts (unculturable) and free-living (culturable) members of the genus to investigate the metabolic potential, genome composition and virulence factors of a novel strain, *Candidatus* Erwinia impunctatus (Erwimp), identified in the pest species of biting midge, *Culicoides impunctatus* (Diptera: Ceratopogonidae). Present across most of Northern Europe, these small blood- and nectar-feeding insects are particularly notable in the Western Scottish highlands where huge numbers form likely as a result of their ability to reproduce successfully once without a blood-meal (autogeny) [19]. Their sheer numbers, as well as multiplicity of ecological interactions (adults feed on both animals and plants), mean that elucidating the relationship between *C. impunctatus* and Erwimp could indicate impacts on the insect itself or the flora and fauna they interact with.

## Methods

### Specimen collection, DNA extraction, genome assembly and annotation

During an attempt to sequence the complete genome of a common *Rickettsia* endosymbiont in *Culicoides impunctatus* [20], a circular contig with homology to Erwiniaceae bacteria was also assembled. This was achieved by the following: five hundred and thirty-one live female *Culicoides impunctatus* were collected by aspiration from the author’s skin in Kinlochleven, Scotland (56° 42′ 50.7″ N 4° 57′ 34.9″ W) in September 2020. Four-hundred and eighty *C. impunctatus* individuals were pooled, while the other 51 were kept for individual screening for any subsequent bacteria identified through sequencing. These were then homogenised and high-molecular weight DNA was extracted using the method described in Davison *et al*. [20]. Oxford Nanopore libraries were generated using the SQK-LSK109 Ligation Sequencing Kit and sequenced on a Minion R9.4.1 flow cell. Raw reads were base called using Guppy version 4.0.15 (Oxford Nanopore) and the high-accuracy (hac) option. All reads >500 bp in length which had an average phred (Q) score of above 10 were filtered using NanoFilt v2.7.180 [21]. Assembly of these reads took place using Flye v2.8.181 with default options [22].

High quality short-read libraries were also generated and sequenced from the same DNA samples using a Kapa HyperPrep kit (Roche) and a DNBseq G50 platform by BGI Genomics (Hong Kong) to correct the initial assembly. Filtering of raw DNBseq reads was performed by BGI Genomics by removing adapters using SOAPnuke v2.1.482 [23] and keeping reads with a mean Q score of >20. Filtered reads were assembled using MEGAHIT v1.2.983 [24] before binning contigs with MetaBAT 2 v2.12.184 [25]. The identities of bins were checked with CheckM v1.1.378 [26] (File S1, available in the online version of this article) before reads allocated to Erwiniaceae were mapped back to the Flye-assembled circular contig using ‘perfect mode’ in BBMap v38.8785 [27] and filtered using SAMtools v1.1186 [28]. These mapped reads were then used to polish SNPs and indels in the initial nanopore assembly using two rounds with Pilon v1.2387 and the *--fix bases* option [29]. Completeness was assessed using BUSCO v5.1.2 [30] through the gVolante server [31] based on 366 single‐copy bacterial markers for gammaproteobacteria (gammaproteobacteria odb10 database). Annotation of the final polished genome was undertaken using PROKKA v1.1388 [32] with identification of secondary metabolites using antiSMASH v7.0 [33].

### Phylogenomics and ANI

As the provisional taxonomic assignment for a ~3.5 Mbp circular contig was as a member of the Erwiniaceae (File S2), a total of 48 genomes from this family spanning the genera *Erwinia*, *Pantoea*, *Tatumella*, *Izakhiella* and *Mixta* (as well as four outgroup genomes; *Pectobacterium brasiliense*, *Serratia marcescens*, *Klebsiella pneumoniae* and *Shigella dysenteriae*) were downloaded from NCBI for further phylogenomic assessment (File S1). To identify and extract single-copy orthologues, anvi’o v7 [34] was used to create a pangenome database. One-hundred and thirty-five single-copy orthologues with a minimum geometric identity of 90 % (to remove poorly aligned genes) from all genomes were then filtered. The *anvi-get-sequences-for-gene-clusters* programme was then used with the *--concatenate-gene-clusters* option to extract the 135 concatenated orthologues for each genome. Gblocks v0.91b [35] was then used to exclude areas of the alignment with excessive gaps or poor alignment. ModelFinder [36] then determined the LG+I+G4 model to be used after selection using the Bayesian information criteria. A maximum likelihood (ML) phylogeny was then estimated with IQTree v2.1.2 [37] using an alignment of 41 012 amino acids and 1000 ultrafast bootstraps. To supplement the phylogenomic inferences, an average nucleotide identity (ANI) was also ascertained using the *anvi-compute-genome-similarity* programme and pyani [38].

### Clusters of orthologous genes (COG), pseudogene and metabolic profiling

To compare the genomic profiles of free-living (culturable) and endosymbiotic (unculturable) *Erwinia* with Erwimp, Clusters of Orthologous Genes (COG) profiles were created using the *anvi-run-ncbi-cogs* programme before the proportions of each category were plotted as a bar plot in ggplot2 v3.4.2 [39] using RStudio v2022.02.0 [40]. Pseudofinder v1.1.0 [41] and DefenseFinder [42] were used to identify the proportion of pseudogenes and number of antiviral defence systems in all *Erwinia* genomes. To compare metabolic profiles between *Erwinia* genomes in the pangenome database, anvi’o was used with the programmes *anvi-run-kegg-kofams* and *anvi-estimate-metabolism*, which utilised previous annotation of genes with KEGG orthologs (KOs) [43]. The outputted completeness matrix for amino acid, lipid, carbohydrate, and vitamin metabolic pathways was then inputted into pHeatmap v1.0.12 [44] and visualised using RStudio. In addition, PhyloFlash v3.4 [45] was utilised to search for SSU rRNAs possibly associated as other sources of Erwimp within the metagenome.

### Virulence factors and plant gene identification

To gain a better understanding of the ecological impact of Erwimp, common virulence factors [46] and plant-symbiosis genes [47] identified previously were compiled and searched for using PathwayTools v26.5 [48]. Presence/absence matrices were created for each of these pathways before being plotted using pHeatmap. Proksee [49] was then used to visualise the circular chromosome of Erwimp and ORFs found in PathwayTools were then annotated in Inkscape v1.1 [50]. Clinker [51] plotted the similarity of carotenoid biosynthetic genes discovered using antiSMASH. Interpro v95.0 [52] provided functional analysis by predicting domains for Intimin-like genes.

### Targeted *Erwinia* screening of *Culicoides impunctatus* individuals

To understand the distribution of Erwimp in *C. impunctatus* populations, a remaining 51 individuals from the 2020 (Kinlochleven) catch, alongside 64 individuals caught in 2021 in neighbouring areas to the initial catch, were stored in 75 % ethanol at −20 °C before individual DNA extracting as described previously [53]. Screening of the cytochrome c oxidase subunit I (*COI*) gene was initially assessed as a means of quality control of extracts by conventional PCR [54]. DNA extracts which passed quality control were then screened using a conventional PCR with primers based on the DNA gyrase subunit B (*GyrB*) gene (Erwimp_Gyrb_1832F: 5′-ATCGTCCGGTATTGTCTGCG-3′; Erwimp_Gyrb_2194R 5′-ATCCACGACGAGACTCCTT-3′). PCR assays consisted of a total of 15 µl per well, comprising of 7.5 µl GoTaq Hot Start Polymerase, 5.1 µl nuclease free water, 0.45 µl forward and reverse primers (concentration 10 pmol µl^−1^) and 1.5 µl DNA template. Cycling conditions were as follows: initial denaturation at 95 °C for 5 min, followed by 35 cycles of denaturation (94 °C, 30 s), annealing (55 °C, 30 s), extension (72 °C, 90 s), and a final extension at 72 °C for 7 min. PCR products were separated on 1 % agarose gels stained with Midori Green Nucleic Acid Staining Solution before UV transillumination. Mapping of results to geographic location was undertaken using QGIS v3.28.2 [55].

## Results

### General features of Erwimp genome

The assembled chromosome of Erwimp is 3 461 605 bp in size with an average GC content of 48.2 % (File S1) and an average depth of coverage of 459×. A BUSCO completeness score of 99.7 % (Single-copy: 99.7 %, Duplicated: 0 %, Fragmented: 0.3 %, Missing: 0 %) suggests a high-quality genome. Genome annotation found 3418 protein coding sequences (CDSs) with an average length of 864 bp, seven copies each of the 5S, 16S and 23S rRNA genes and 69 tRNA genes with a coding density of 85.3 %. Of the 3418 predicted CDSs, 2472 (~72 %) were annotated with putative functions, while 946 (~28 %) CDSs were annotated as hypothetical proteins. In addition, one 134kbp plasmid (pErwimp001) was also putatively identified.

**Fig. 1.**
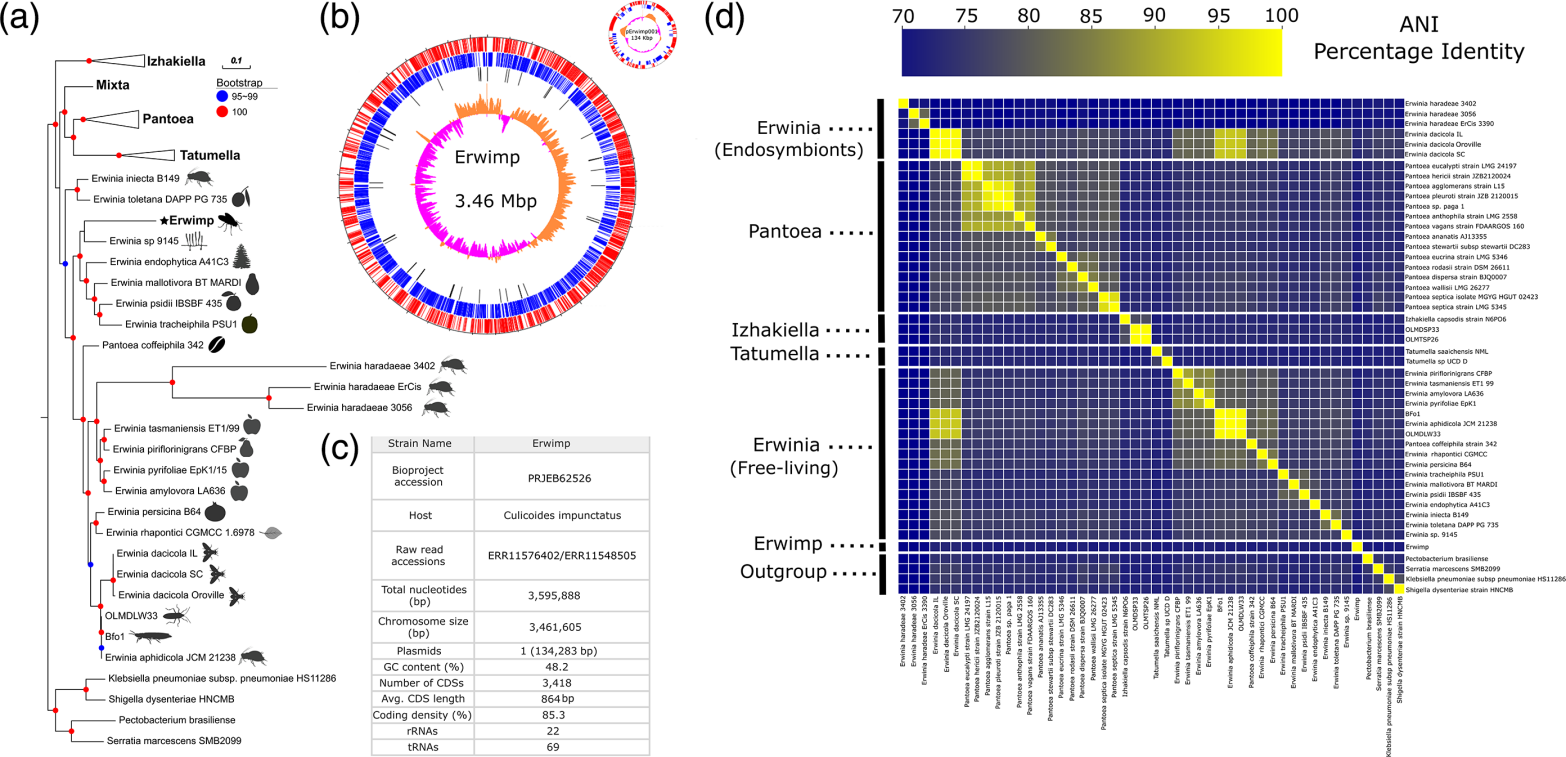
(**a**) Maximum-likelihood phylogenomic tree of the Erwiniaceae family based on 135 concatenated single-copy orthologues. Leaf silhouettes represent the isolation source of *Erwinia* spp. strains. The Erwimp isolate described in this study is denoted in bold and a star symbol. (**b**) A genome plot of Erwimp. From outermost rings inward, CDSs on the direct strand, CDSs on the reverse strand, tRNAs, G-C skew. (**c**) Genome features and project accession information. (d) Average Nucleotide Identity (ANI) heatmap of Erwiniaceae genomes.

Through an initial phylogenetic screen of five concatenated house-keeping genes, the bacterial chromosome appeared to cluster within the *Erwinia* genus of the family (File S2). Subsequently, to confirm this as a new species member of the group, the phylogenomic relationship of Erwimp relative to other Erwiniaceae was estimated (Fig. 1a) from a set of 144 single-copy orthologues identified among 44 draft or complete genomes (23 *Erwinia*, 15 *Pantoea*, three *Izhakiella*, two *Tatumella* and one *Mixta*). The maximum-likelihood tree placed Erwimp within a subclade of the genus incorporating *Erwinia tracheiphila*, *Erwinia psidii*, *Erwinia mallotivora* and *Erwinia endophytica*, with the most recent known ancestor being *Erwinia* sp.1945, an endohyphal bacteria of *Microdiplodia* sp. As an adjunct to the phylogenomic data, Average Nucleotide Identity (ANI) was compared between all genomes. The closest percentage identity of any genome to Erwimp was 74 % clearly delineating it as a new species (Fig. 1d).

### COG, metabolic, pseudogene and defense system profiles

**Fig. 2.**
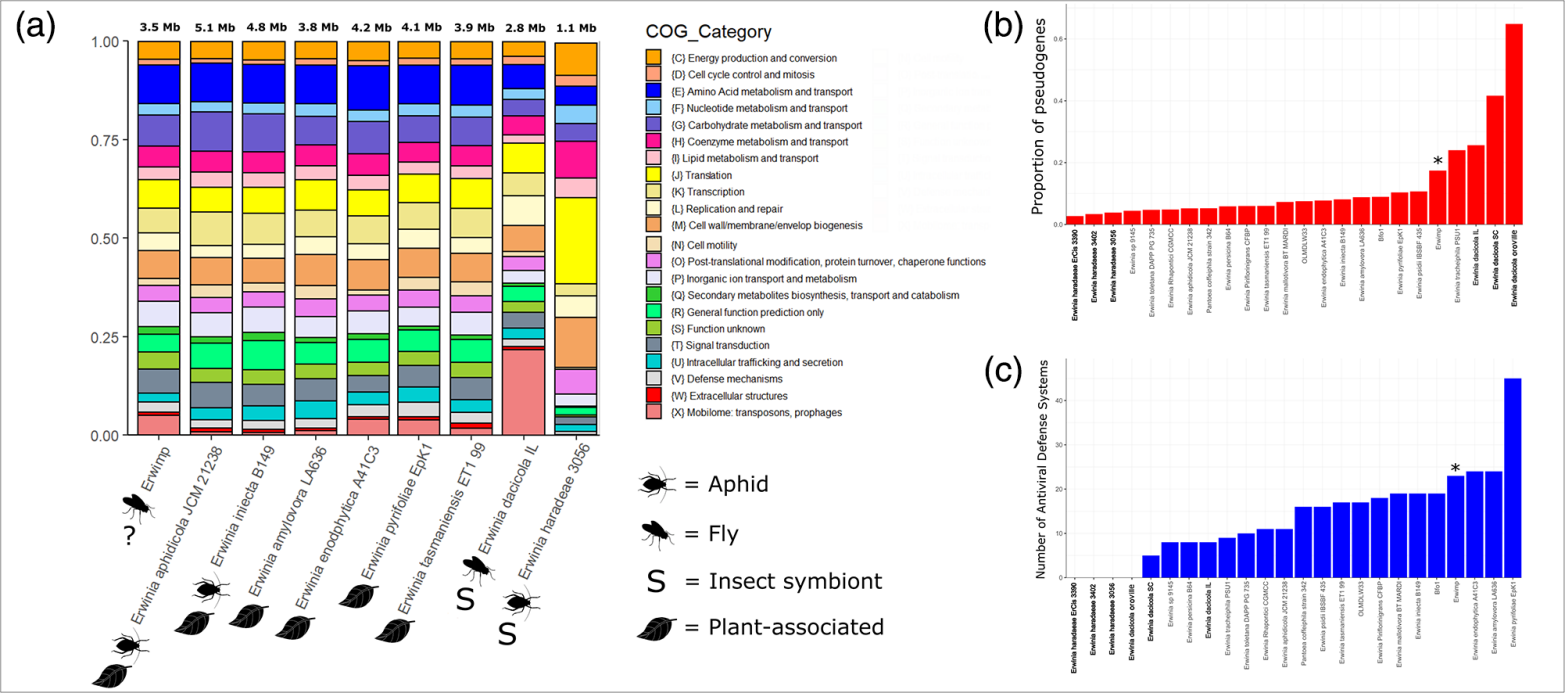
(**a**) Frequencies of Clusters of Orthologous Genes (COG) clusters for Erwimp compared to a subset of free-living *Erwinia* strains and insect endosymbionts. (**b**) The proportion of pseudogenes and number of bacterial defence systems (**c**) attributed to each *Erwinia* species. For **b** and **c** bold species names indicate endosymbiont strains.

Insect endosymbionts and free-living bacteria have varying distributions of functional gene categories as a result of different selection pressures. To provide insights into the lifestyle of Erwimp, its Clusters of Orthologous Genes (COG) profile was compared to other *Erwinia* (Fig. 2a). Generally, Erwimp matched the profiles of both endophytic and epiphytic species with increased relative proportions compared to insect endosymbionts of amino acid metabolism and transport (COG category E), signal transduction mechanisms (T), inorganic ion transport (P), and carbohydrate metabolism and transport (G) gene categories. Furthermore, the 5 % proportion of Erwimp mobilome genes (X) is comparable to the free-living *Erwinia pyrifoliae* (4 %) and *Erwinia endophytica* (4 %) but much lower than *Erwinia dacicola* (21 %) and higher than *Erwinia haradaeae* (0.1 %) endosymbionts.

**Fig. 3.**
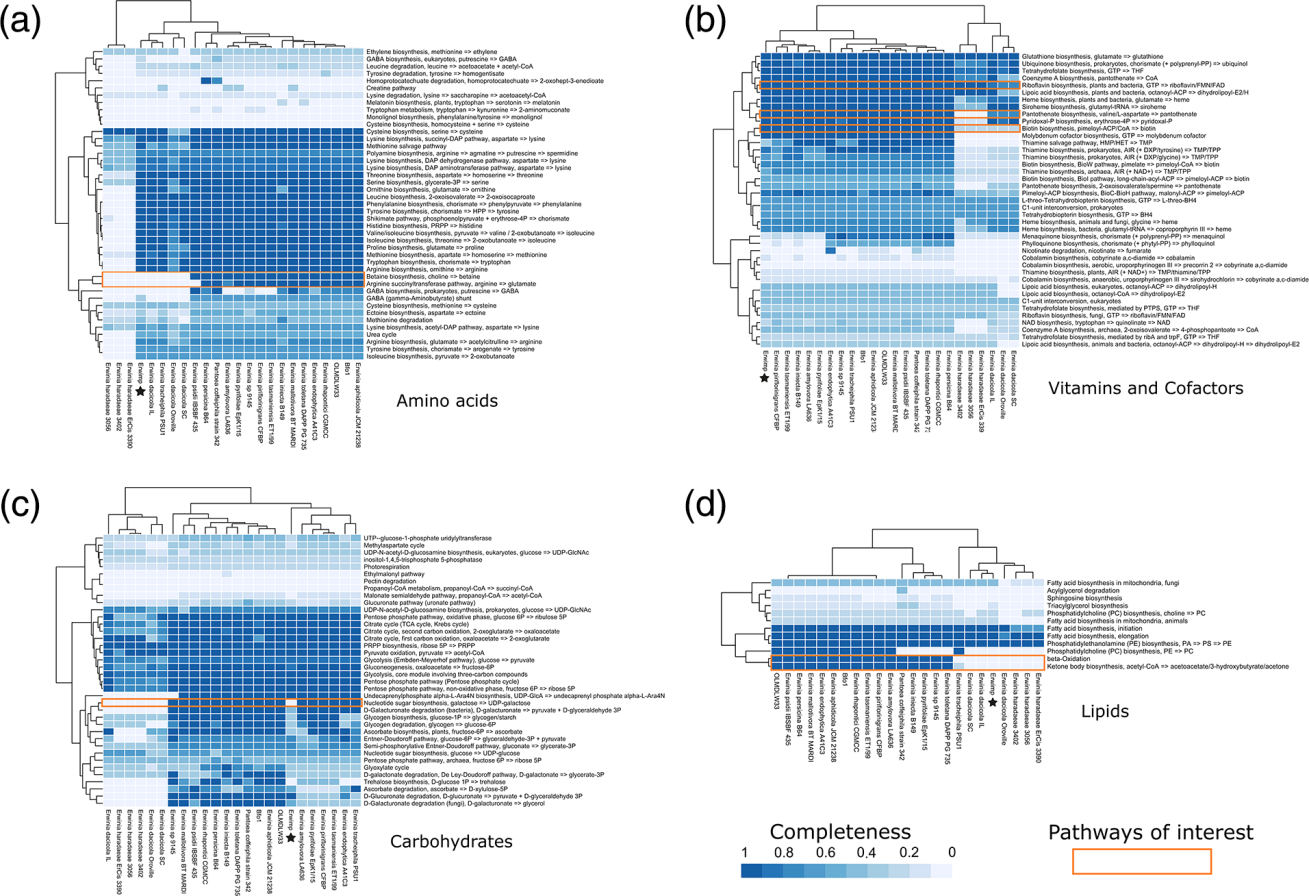
Metabolic profile heatmaps of predicted Kyoto Encyclopaedia of Genes and Genomes (KEGG) pathways. Erwimp metabolic profiles are denoted by stars. (**a**) Amino acid metabolism. (**b**) Vitamins and cofactors. (**c**) Carbohydrate metabolism. (**d**) Lipid metabolism.

Erwimp’s metabolic potential (Fig. 3) is somewhat similar to members of the genus which have undergone genome decay as a result of host specialisation (e.g. *Erwinia tracheiphila*, *Erwinia dacicola* and *Erwinia haradaeae*). For example, amino acid biosynthesis pathways for glutamate and betaine are absent for Erwimp, *Erwinia tracheiphila*, *Erwinia dacicola* and *Erwinia haradaeae*, but are generally complete elsewhere (Fig. 3a). The same pattern is observed for lipid metabolism with absent pathways for beta-oxidation in acyl-coA degradation as well as ketone body synthesis (Fig. 3d). Erwimp also lacks the capability of producing UDP-galactose, a key component of nucleotide sugar metabolism (Fig. 3c). Further evidence of relative genome degradation comes from the proportion of pseudogenes in the Erwimp genome (17.4 %), which is higher than most free-living (mean=8.0 % [95 % CI: 7.0–9.0 %]) species although slightly lower than those thought to have undergone host-specialisation (e.g. *Erwinia tracheiphila* [24 %]; Fig. 2b). Co-factors and vitamin pathways were also assessed (Fig. 3b) due to both plant and blood-sucking insect bacteria assisting in the provisioning of B-vitamins often lacking in the host’s diet. Erwimp contains the complete pathways for pantothenate, biotin and riboflavin biosynthesis although this does not appear to be unique across the genus, with all free-living species also containing these pathways, but not endosymbionts (apart from *Erwinia haradaeae* and riboflavin). A total of 23 antiviral defence systems were identified in Erwimp compared to a mean of 17.1 (95 % CI: 13.19–19.29) and 2.6 (95 % CI: 0–4.7) for free-living and endosymbiont strains respectively.

### Genetic repertoire of Erwimp

**Fig. 4.**
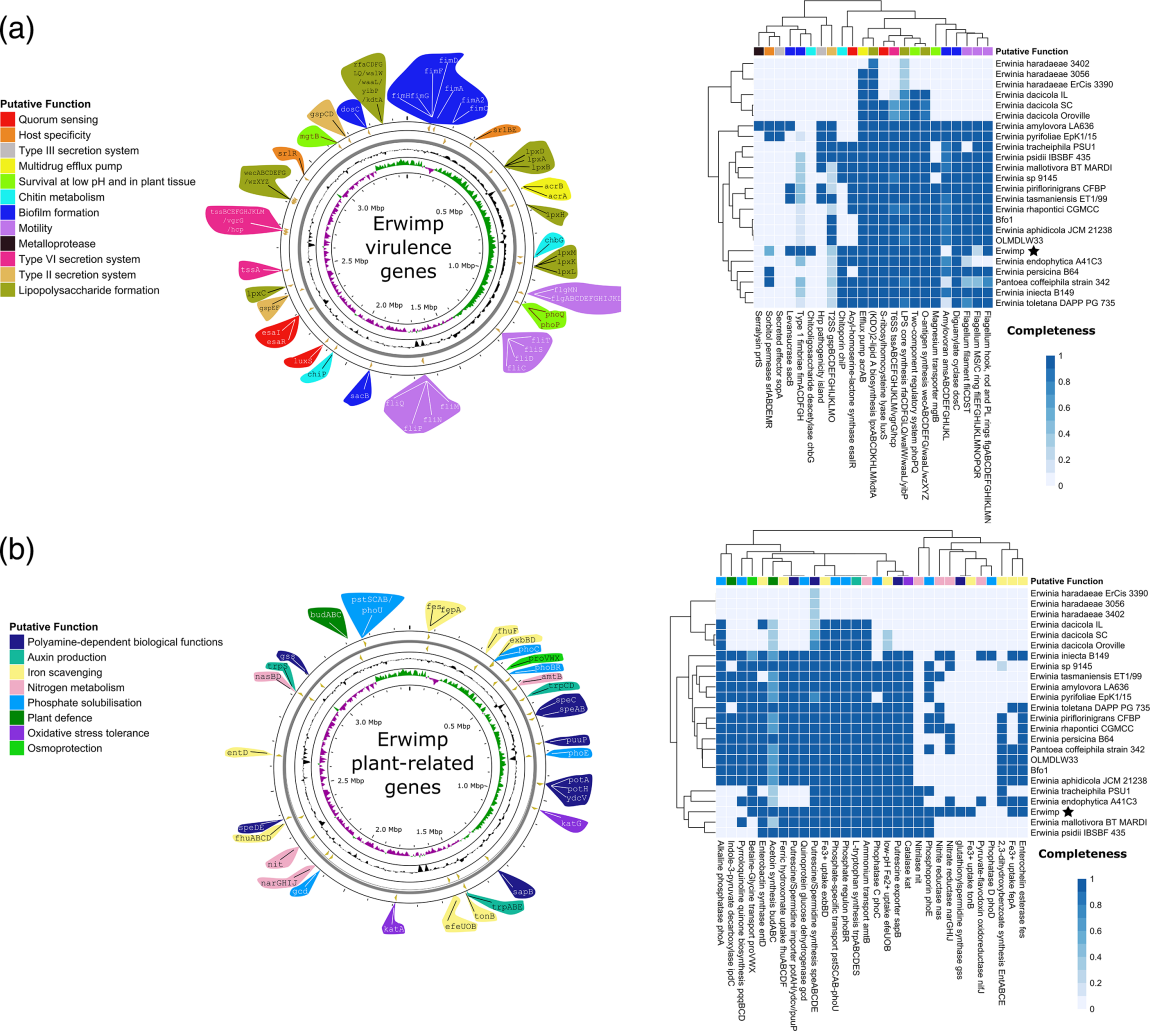
Annotated Erwimp chromosomes detailing common *Erwinia* virulence factors (**a**) and putative plant-related genes (**b**) alongside their completeness in other *Erwinia* species.

The presence and absence of common *Erwinia* virulence factors were compared among all strains (Fig. 4a). Erwimp appears to hold the capacity to communicate through quorum sensing (QS), containing the QS regulatory system *EsaI*/*EsaR,* as well as s-ribosylhomocysteine lyase (*LuxS*) which synthesises autoinducer-2. Additionally, the genome contains a full type 1 fimbrial gene cluster which is not present in any other members of the genus assessed. Out of the characterised *Erwinia* biofilm exopolysaccharides, amylovoran and levan, the latter but not former is predicted to be produced by Erwimp. Furthermore, a type VI secretion system spanning across two loci is present although other secretion systems commonly found across the genus (types II and III) are incomplete or absent. Flagellar genes encoding the filament, rod, hook and P/L ring structures are present, but only *FliMN* genes of the motor apparatus exist (missing basal MS and C ring components) suggesting an absent or imperfect functioning motility system. Intriguingly, a unique Erwimp gene identified is a chitooligosaccharide deacetylase (*ChbG*) homolog. *ChbG* hydrolyses the N-acetyl group at the reducing-end of chitin disaccharides and is complemented by the presence of a putative chitoporin transport channel (*ChiP*). Finally, a full complement of lipopolysaccharide (LPS) genes (Lipid A, core and O-antigen) are present in most free-living *Erwinia*, as well as Erwimp.

Plant-related genes were also assessed to clarify potential plant-growth and protection attributes (Fig. 4b). Unlike *Erwinia* endosymbionts, Erwimp contains all necessary genes to produce (*SpeABCDE*) and import/export spermidine (*PuuP, SapB, PotA, PotH, YdcV*) suggesting a role in polyamine-related processes such as swarming in bacteria and potentially plant defence responses and abiotic stress tolerance. Additionally, osmoprotectant (betaine glycine transporter; *ProVWX*) and antioxidant (Catalase; *Kat*) genes present exclusively in free-living plant *Erwinia* are also found in Erwimp. Furthermore, a biosynthetic gene cluster for the plant-protecting volatile organic compound acetoin (*BudABC*) is present.

Regarding the capacity of Erwimp to mobilise nutrients for potential plant hosts, Erwimp appears to have lost key genes relating to phosphate solubilisation present in other plant-associated *Erwinia*. These include genes producing pyrroloquinoline quinone (*PqqBCD*), a cofactor for quinoprotein glucose dehydrogenase (*Gcd*), enabling the solubilisation of inorganic phosphorous. Partial pathways related to other plant physiological processes are found such as the complete split operon for the biosynthesis of l-tryptophan (*TrpABCDES*), a precursor of the phytohormone indole-3-acetic acid (IAA). However, the ipdC pathway converting l-tryptophan to IAA is absent in Erwimp. Iron scavenging genes are also found in the genome. Specifically, the chromosome contains the enterobactin synthase gene, *EntD*, enabling the final step in enterobactin synthesis, but lacks the ability to produce the upstream precursor 2,3-dihydroxybenzoic acid. A desferrioxamine transport system (*FhuABCDF*), responsible for the sequestering of ferric hydroxamate siderophores is also present. With respect to nitrogen metabolism, Erwimp nitrate/nitrite reductases (*NarGHI*, *NasBD*) and a nitrilase (*Nit*) suggest potential roles in plant nitrogen assimilation but the bacterium lacks nitrogen fixing pathways.

### Carotenoid and Intimin-like genes

**Fig. 5.**
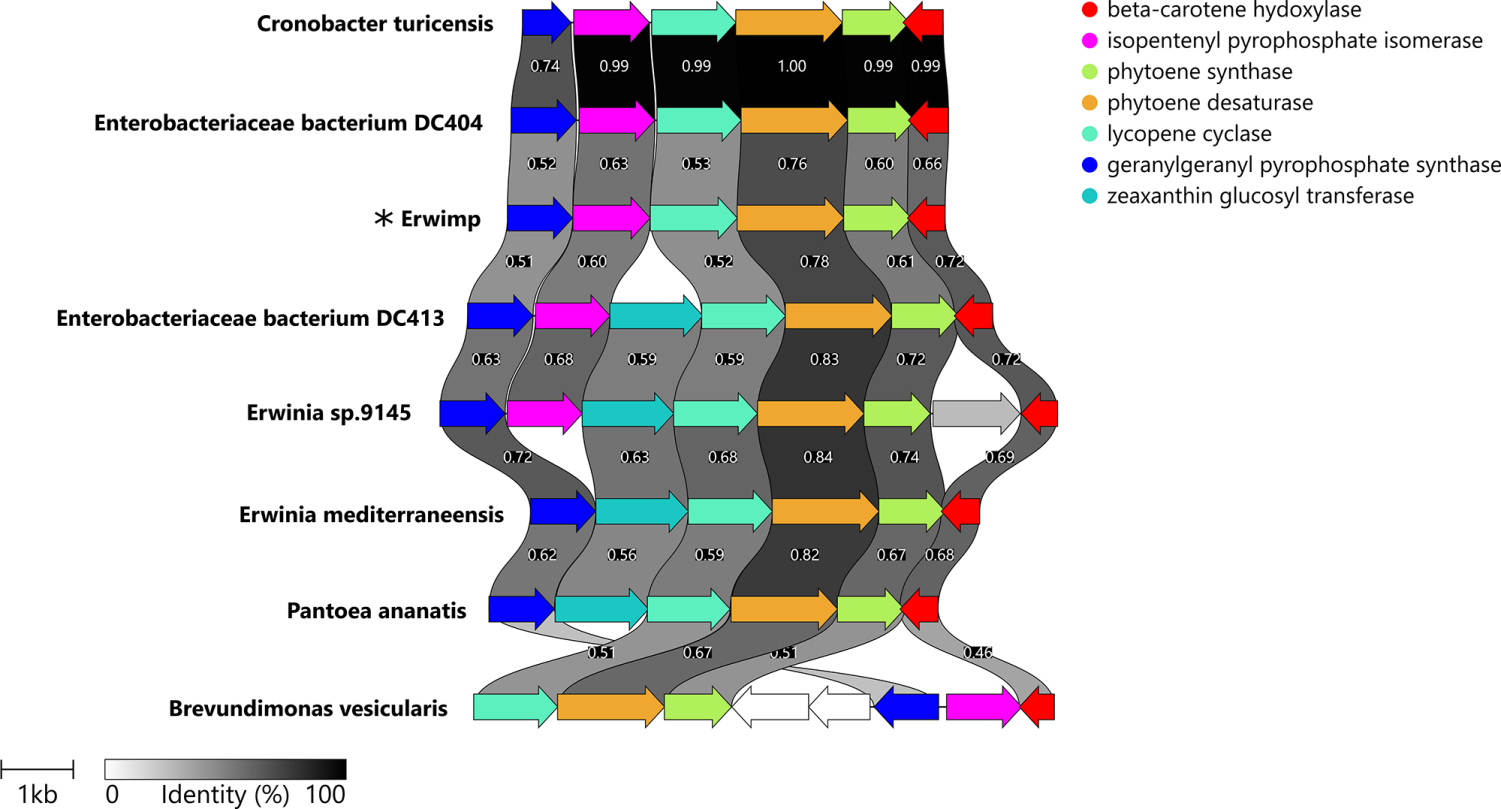
Clinker plot used to compare and visualise the similarity of the Erwimp carotenoid biosynthesis operon uncovered by antiSMASH with other gene clusters based on an all-vs-all similarity matrix.

Secondary metabolite analysis identified the presence of a carotenoid biosynthetic gene cluster (Fig. 5). Only two other *Erwinia* genomes assessed for secondary metabolites were predicted to contain similar operons (File S1). Of interest, is the loss of zeaxanthin glucosyltransferase (*CrtX*) within Erwimp’s gene cluster compared to its nearest known relative *Erwinia* sp. 1945. *CrtX* is necessary for the synthesis of zeaxanthin glucosides and suggests Erwimp is only able to synthesise the simpler linear zeaxanthin molecule.

**Fig. 6.**
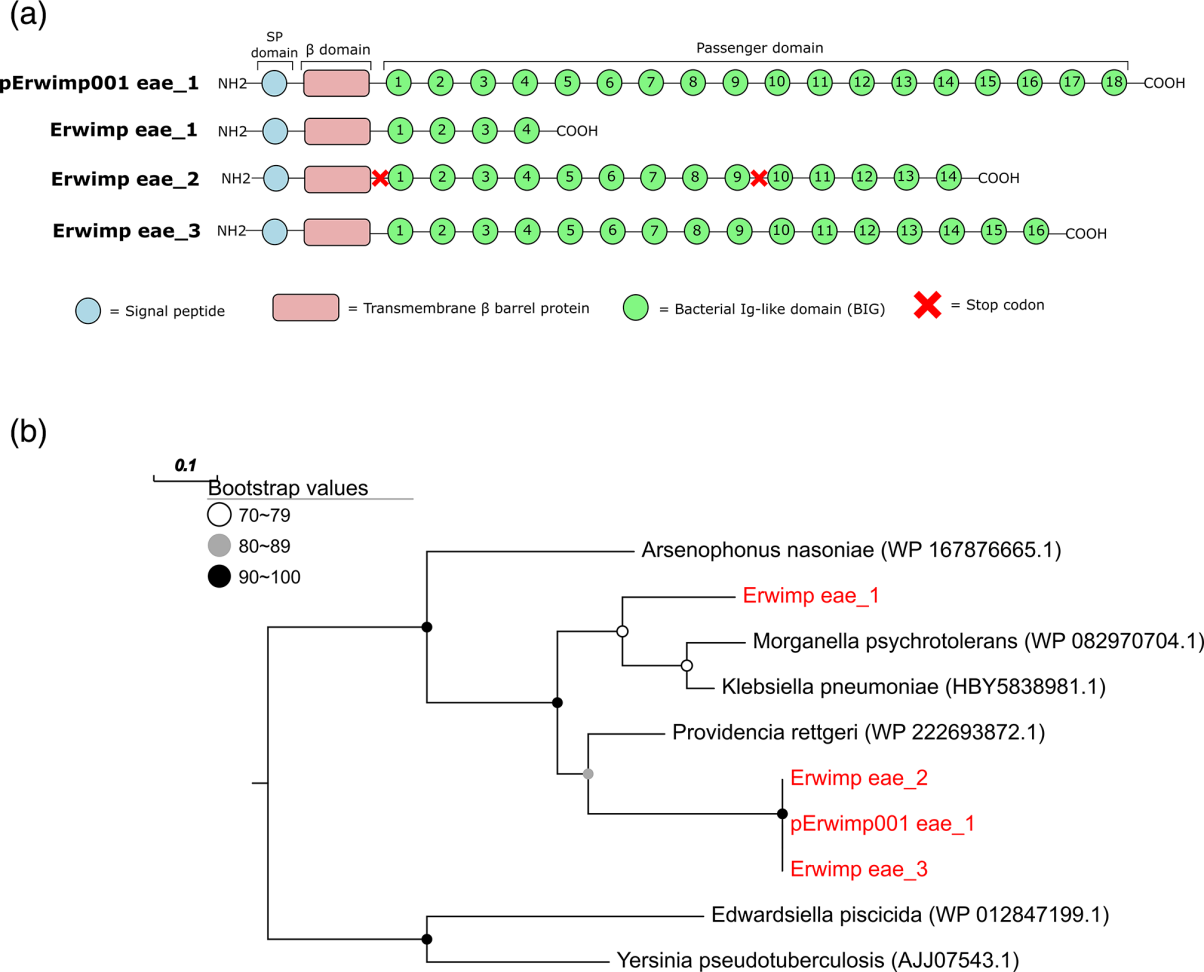
(**a**) Interpro predictions of domains found in multiple Intimin-like proteins found on an Erwimp plasmid (pErwimp001_eae_1) and chromosome (Erwimp_eae_1, Erwimp_eae_1, Erwimp_eae_1). (**b**) Maximum-likelihood phylogeny of the beta-domain from intimin copies based on a 240 amino acid alignment.

A putative 134kbp Erwimp plasmid (pErwimp001) was also assembled and annotated (File S2). pErwimp001 has copies of notable genes present on the chromosome including a putrescine importer (*PuuP*), an acyl-homoserine lactone synthase/repressor (*EsaI/EsaR*) and fimbriae genes (*FimC, FimD*). In addition, an ectoine dioxygenase (*EctD*), catalysing the production of the desiccant protectant hydroxyectoine is present on pErwimp001 but absent in all other *Erwinia* genomes analysed, with the nearest hit being to *Izhakiella capsodis* (WP_092877528.1; 77 % identity). Intriguingly, the largest gene on the plasmid (*eae*; 3213 amino acid residues) is an intimin homolog. Three copies of this gene are present on the Erwimp chromosome with a varying number of bacterial immunoglobulin-like domain (BIG) repeats at the C-terminus (Fig. 6a). Two of these (Erwimp_eae_1 and Erwimp_eae_3) contain all three intimin domains (signal peptide, transmembrane β-barrel and passenger domains) as an operon, whereas Erwimp_eae_2 contains two stop codons between β and passenger domains suggesting pseudogenization. Despite the difficulty in assigning plasmids to particular chromosomes in metagenomic data, the presence of near identical intimin homologues on both pErwimp001 and Erwimp suggests both are likely part of the same genome. No other *Erwinia* genomes in the comparative analysis are predicted to contain intimins and the close identity of pErwimp001_eae_1, Erwimp_eae_2 and Erwimp_eae_3 (>97 % identity over 2664 amino acid residues) suggests a recent plasmid integration involving the Erwimp chromosome followed by gene duplication. A phylogeny of the conserved β domain suggests Erwimp_eae_1 has a separate evolutionary origin to the other three intimins (Fig. 6b).

### Targeted screening of *Culicoides impunctatus* individuals

**Fig. 7.**
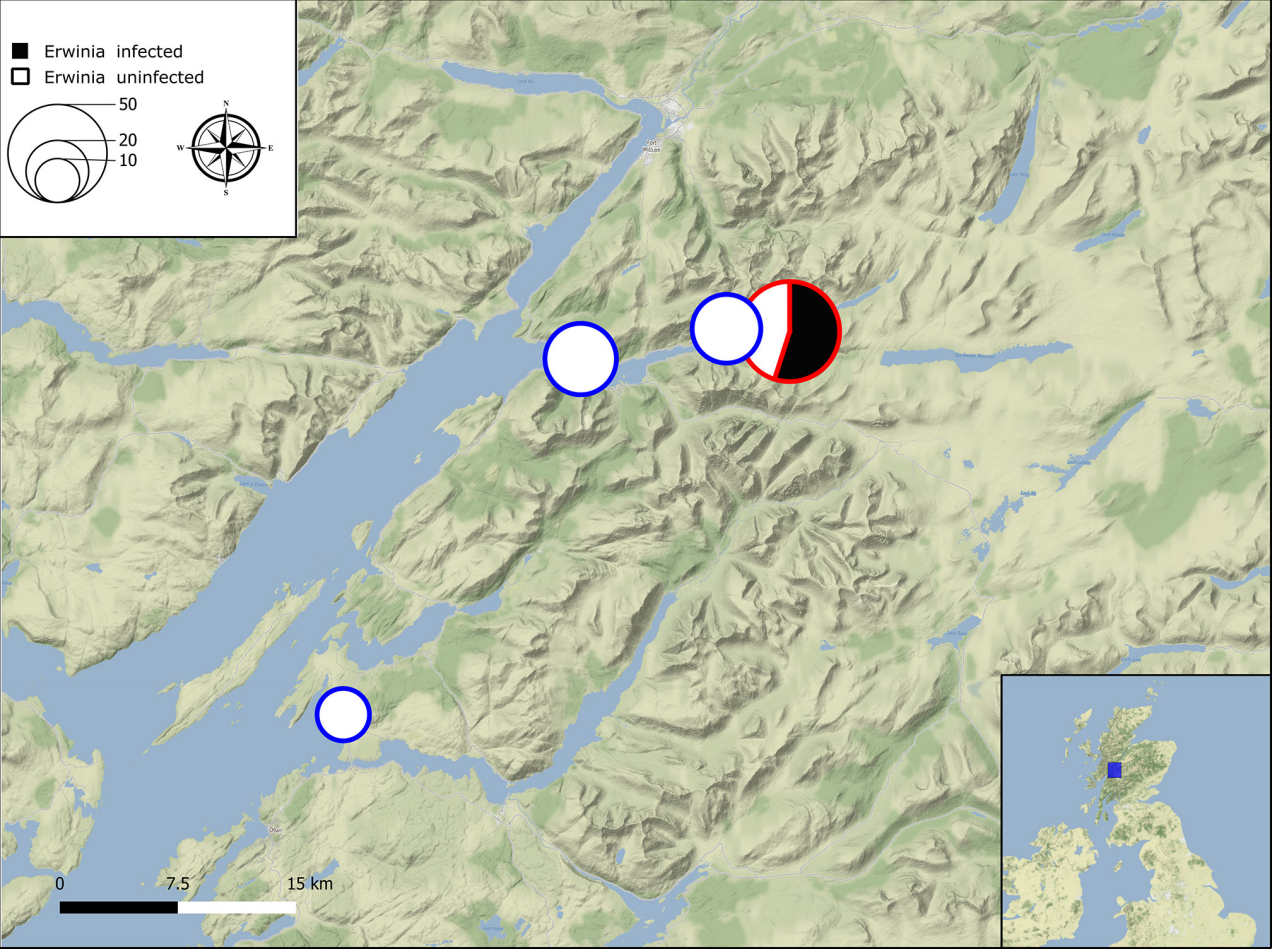
A QGIS map of the Western Scottish Highlands depicting catch sites for *Culicoides impunctatus* during June 2020 (red) and July 2021 (blue) for the targeted PCR screening of *Erwinia*. Size of circles indicate number of specimens per site with the black portion of a circle showing the number of PCR positives.

A total of 115 *Culicoides impunctatus* individuals were screened for the presence of *Erwinia*. Of 51 insects from the 2020 catch, 28 (55 %) were positive by conventional PCR. Returning the following year, no infected insects were identified at the same site as well as neighbouring locations of the 64 screened (Fig. 7).

### Metagenome assessment of potential Erwimp hosts

Aside from *Culicoides impunctatus*, SSU rRNAs exploration of the BGI-derived metagenomic raw-read data provided evidence for one potential plant host, *Arabidopsis thaliana* (Brassicaceae) (File S1). Additional eukaryotes found in the dataset include the fungi *Entomophthora culicis* (Entomophthoraceae) and *Kluyveromyces lactis* (Saccharomycetaceae).

## Discussion

Interactions between microbes and their insect hosts can be transitory or sustained depending on their functional relationship [1,56]. In the case of *Erwinia*, members of the genus have evolved to fill various ecological niches including as insect endosymbionts and phytopathogens [10,15, 57]. Thus, the discovery of Erwimp in *Culicoides impunctatus* raises questions as to the nature of this association.

The functional gene categories of Erwimp are generally comparable to endophytic and epiphytic members of the genus including increased gene frequencies relating to carbohydrate and amino acid metabolism compared to insect endosymbionts (see also [58]). A further intriguing difference between free-living and unculturable insect endosymbionts is the gene frequencies of mobile genetic elements (MGEs). Newton and Bordenstein [59] demonstrated that the largest numbers of MGEs are found in facultative intracellular bacteria (e.g. *Erwinia dacicola*), followed by obligate extracellular (e.g. *Erwinia pyrifoliae*) and obligate intracellular bacteria (e.g. *Erwinia haradaeae*). This pattern is observed in the current study with the mobilome profiles of Erwimp being comparable to the plant-associated *Erwinia pyrifoliae* and *Erwinia endophytica* but being much lower than *Erwinia dacicola* and higher than *Erwinia haradaeae* endosymbionts respectively. Furthermore, extracellular bacteria have a relatively larger number of antiviral systems compared to endosymbionts [42] possibly as they are more likely to encounter phages. The number of defence systems identified in Erwimp aligns with other free-living members of the genus (Fig. 1c) and gives further support for an obligate extracellular lifestyle. Finally, virulence factors specific to free-living *Erwinia* tend to be found in Erwimp, many of which are likely directly or indirectly related to biofilm formation. These include the ability to produce the exopolysaccharide (EPS) levan [60], a type VI secretion system associated with EPS production and interbacterial competition [61], a lipopolysaccharide (LPS), quorum sensing genes and a full type one fimbrial gene cluster.

The frequent transmission of *Erwinia* to plant hosts via insect vectors [13,14] suggests local vegetation should be considered as additional putative hosts of Erwimp. Potential plant hosts include *Arabidopsis thaliana* which occurs predominantly in the Scottish highlands during the summer months [62] at the same time as midge season and was found as the only plant SSU RNA detected in the metagenomic data (File S1). Further hosts to consider include those part of *C. impunctatus’* larval/pupal habitats, such as *Sphagnum* spp. and *Polytrichum* spp. mosses [63], as well as adult resting sites like downy birch (*Betula pubescens*) trees [64]. Homologs of putative plant growth promoting and defence genes were observed in the Erwimp genome (Fig. 4b), however, these genes often included those with an ambiguous ontology. For example, spermidine/putrescine synthesis and transport genes (*speABCDE/puuP, sapB, potA, potH, ydcV*) could be related to host or bacterial polyamine-related functions such as abiotic stress tolerance [65] in the former and chemotaxis or growth in the latter [66]. Additionally, genes related to nitrogen assimilation, siderophore synthesis and phosphate mobilization are present but Erwimp still lacks full pathways for enterobactin synthesis, nitrogen fixation, IAA synthesis and phosphate solubilization by gluconic acid.

Despite this, a notable carotenoid biosynthetic gene cluster, implicated as a virulence factor in plant pathogens of the sister genus, *Pantoea* [67,69], is also present in Erwimp (Fig. 5). Carotenoids are pigments associated with UV and oxidative stress tolerance in *Pantoea* spp. where they are under regulation by the *EsaI*/*EsaR* quorum-sensing regulatory system [67]. Aside from assisting in photo-tolerance, phytoene synthase (*CrtB*) defective mutants have been shown to impair biofilm formation [68] and root colonization [69] in *Pantoea* sp. YR343. Furthermore, the predicted product of this operon, zeaxanthin, is a precursor to the phytohormone abscisic acid (ABA) [70]. Thus, despite Erwimp’s loss of the idpC pathway for IAA synthesis, assistance in ABA production may still play a role in the growth control of a plant host. Aside from plants, *Erwinia* extends its presence to endohyphal environments such as the case of Erwimp’s closest relative, Erwinia sp.1945 in *Microdiplodia* sp. [16]. The metagenome reveals potential hosts such as the fungal insect pathogen *Entomophthora culicis* and yeast *Kluyveromyces lactis*. Notably, the identification of a chitooligosaccharide deacetylase (*ChbG*), involved in chitin metabolism [71], is absent in all studied *Erwinia* genomes apart from Erwimp hinting at a possible role in a fungal or insect niche.

Regardless of additional Erwimp hosts, the genome’s sizable proportion of pseudogenes (Fig. 2b) along with the loss of certain components of amino acid, carbohydrate, and fatty acid metabolism (Fig. 3) is reminiscent of *Erwinia tracheiphila*. In this case, the cucurbit plant wilt pathogen, has become obligately insect-transmitted leading to genome decay [72] and suggests a possible similar host restriction for Erwimp. Intriguingly, multiple intimin (*eae*) copies unique to *Erwinia* are found within the Erwimp genome. Intimins are a family of outer membrane proteins generally found in the Enterobacterales which act as adhesins [73]. The presence of three near identical intimin-like proteins on the plasmid and chromosome (two intact and one pseudogenized), indicates a recent chromosome integration event, and suggests these are important for host interactions. Aside from offering a further putative mechanism for Erwimp adherence to eukaryotic cells and biofilm formation [74], *in vivo* and *ex vivo* studies investigating the interchange of *E. coli* intimin subtypes [75], found these adherence factors are determinants of host specificity and tissue tropisms offering further evidence for a narrow host range for Erwimp.

Of the culturable *Erwinia* strains associated with insects, several inhabit the gut and have been shown to be sustained over several generations (e.g. BFo1 symbiont of western flower thrips [76]) or are transient associations (e.g. *Erwinia aphidicola* and *Erwinia iniecta* in aphids [77,78]). After the initial identification of Erwimp at intermediate prevalence in the 2020 collection, the acquisition of more infected *C. impunctatus* for biochemical and imaging analysis was attempted the following field season (2021), however, no more Erwimp positive insects were identified. This suggests Erwimp may inhabit *C. impunctatus* transiently, as co-evolved symbioses between gut bacteria and insects are often widespread and at high prevalence in populations [79].

*Culicoides impunctatus* impacts Scottish tourism and forestry industries through its voracious biting of humans and as a hazard to safe arboricultural operations [80]. Given the abundance and importance of *C. impunctatus* as a biting pest, the effects of this bacterium on insect fitness is of great interest. Prokaryotes have been shown to supplement their insect hosts with B-vitamins, particularly those reliant on blood or phloem feeding [6]. Although Erwimp contains the full pathways for the synthesis of several B-vitamins, this is not an exclusive feature amongst other strains indicating this is likely not a symbiotic feature. Experimental assessment of host fitness for several *Erwinia* present in insect guts have provided a spectrum of outcomes from pathogenic to mutualistic [78,81, 82]. However, of the members deemed as pathogens (*Erwinia iniecta*, *Erwinia aphidicola*) experimentation was conducted under laboratory conditions and possibly at unrealistic litres using an artificial diet [78,81]. Similarly, the beneficial effects on development and fecundity observed with BFo1 in western flower thrips is diet-dependent [82]. Overall, the cryptic nature of *Erwinia*-insect interactions suggest further work is required to assess the impact of Erwimp on *Culicoides impunctatus*, beginning with confirming culturability. Furthermore, consideration should be given to the insect vector potential of *C. impunctatus* to transmit Erwimp, as this is likely to have an impact on local vegetation with both plant pathogenicity and growth promotion [83] possible.

## supplementary material

10.1099/mgen.0.001242 Uncited Supplementary Material 1.

10.1099/mgen.0.001242 Uncited Supplementary Material 2.

## Abbreviations

ABA: abscisic acid
ANI: average nucleotide identity
BIG: bacterial immunoglobulin-like domain
CDS: coding sequence
COG: clusters of orthologous genes
eae: Initmin
ENA: European nucleotide archive
Erwimp: Erwinia impunctatus
IAA: indole-3-acetic acid
KEGG: kyoto encyclopaedia of genes and genomes
MGE: mobile genetic element
QS: quorum sensing
