# Supplementary material for: Comparative genomics of a novel Erwinia species associated with the Highland midge (Culicoides impunctatus)
Source: Microb Genom. 2024 Apr 17;10(4):001242. doi: 10.1099/mgen.0.001242 (PMC11092252; doi:10.1099/mgen.0.001242)
Supplement: Uncited Supplementary Material 2. [file mgen-10-01242-s002.pdf]

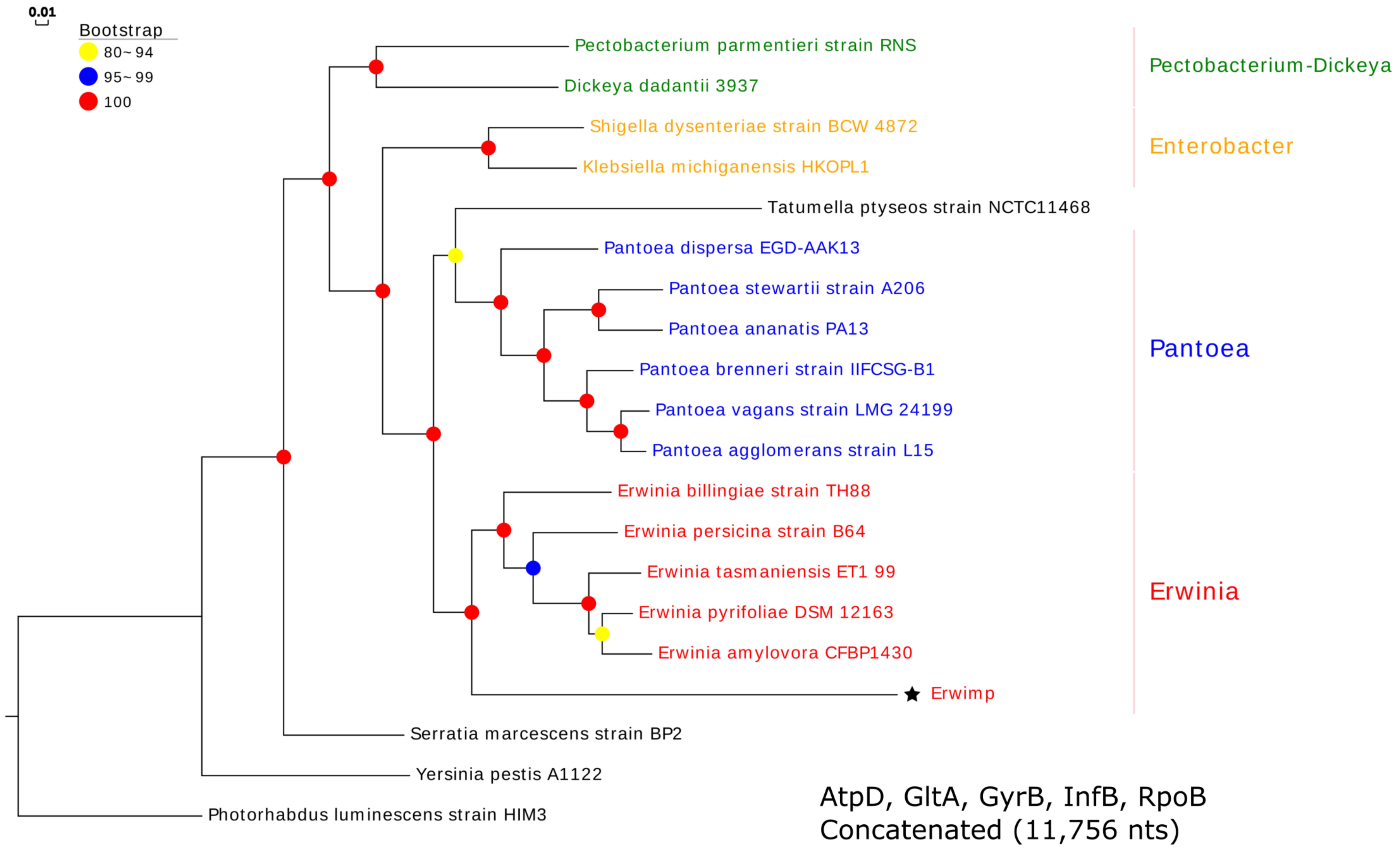
0.01
Bootstrap
80~ 94
95~ 99
100
Pectobacterium parmentieri strain RNS
Dickeya dadantii 3937
Shigella dysenteriae strain BCW 4872
Klebsiella michiganensis HKOPL1
Tatumella ptyseos strain NCTC11468
Pantoea dispersa EGD-AAK13
Pantoea stewartii strain A206
Pantoea ananatis PA13
Pantoea brenneri strain IIFCSG-B1
Pantoea vagans strain LMG 24199
Pantoea agglomerans strain L15
Erwinia billingiae strain TH88
Erwinia persicina strain B64
Erwinia tasmaniensis ET1 99
Erwinia pyrifoliae DSM 12163
Erwinia amylovora CFBP1430
Erwimp
Serratia marcescens strain BP2
Yersinia pestis A1122
Photorhabdus luminescens strain HIM3
Pectobacterium-Dickeya
Enterobacter
Pantoea
Erwinia
AtpD, GltA, GyrB, InfB, RpoB
Concatenated (11,756 nts)

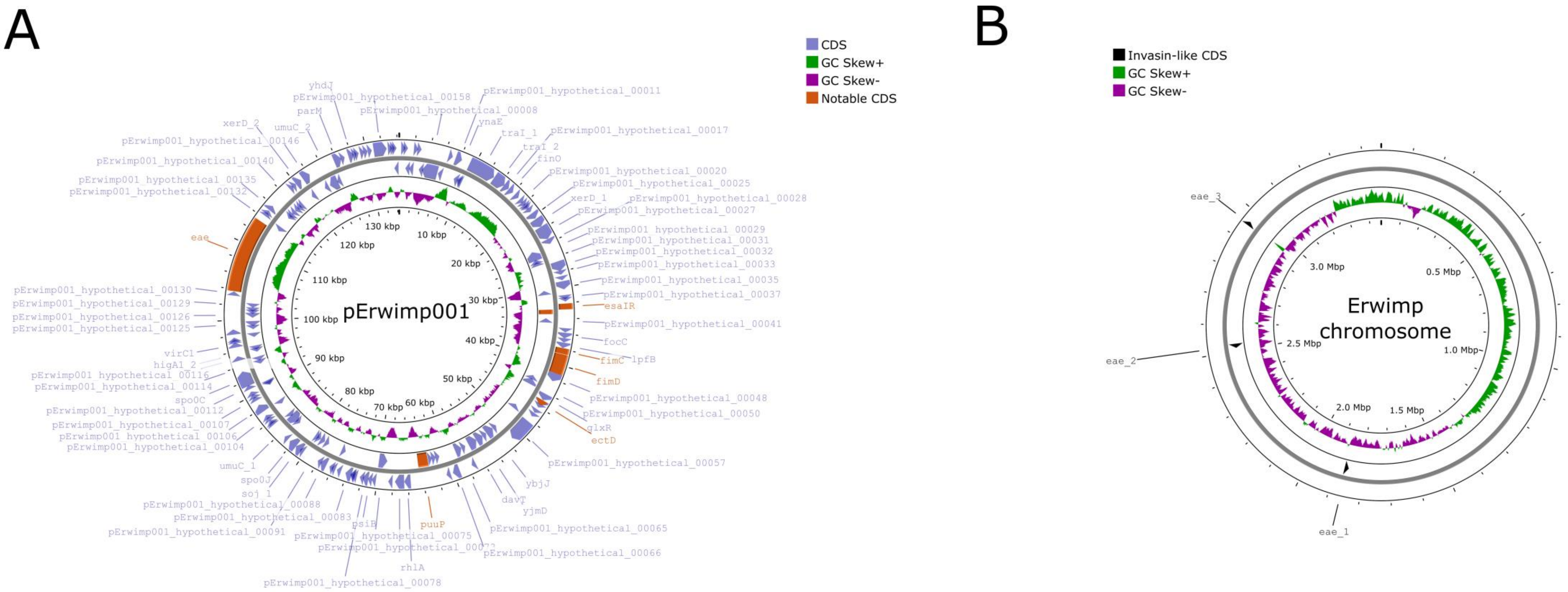

A) Plasmid pErwimp001 annotation. **B)** Location of intimin/invasin-like proteins on Erwimp chromosome.
